# *catena*-Poly[(μ-anilido)(μ-1,2-dimeth­oxy­ethane-κ^3^-*O*,*O*′:*O*)sodium]

**DOI:** 10.1107/S1600536812040500

**Published:** 2012-09-29

**Authors:** Phil Liebing, Christoph Wagner, Kurt Merzweiler

**Affiliations:** a Institut für Chemie, Naturwissenschaftliche Fakultät II, Martin-Luther-Universität Halle-Wittenberg, Kurt-Mothes-Str. 2, 06120 Halle, Germany

## Abstract

In the title compound, [Na(C_6_H_5_NH)(C_4_H_10_O_2_)], the Na^+^ cation is coordinated by the N atoms of two anilide anions, two O atoms of a chelating 1,2-dimeth­oxy­ethane (dme) ligand and one O atom of an adjacent dme ligand. The coordination polyhedron around Na^+^ corresponds to a distorted square pyramid with the N atoms of the anilide groups and the O atoms of the chelating dme unit at the base and a third O atom at the apical position. The anilide anions act as μ-bridging ligands and the 1,2-dimeth­oxy­ethane mol­ecules display a μ_2_-κ^3^-*O*,*O*′ coordination mode. As a result of this connectivity, a polymeric chain structure parallel to [100] is formed, consisting of Na_2_O_2_ and Na_2_N_2_ four-membered rings. It should be noted that the remaining H atom of the anilide NH group is not involved in hydrogen bonding.

## Related literature

For the crystal structure of a sodium anilide complex, see: Barr *et al.* (1995 ▶) and for the crystal structures of sodium compounds with μ-bridging 1,2-dimeth­oxy­ethane ligands, see: Bock *et al.* (1999 ▶, 2000 ▶); Rothenberger *et al.* (2007 ▶); Tirla *et al.* (2002 ▶). For a description of the Cambridge Structural Database, see: Allen (2002 ▶).
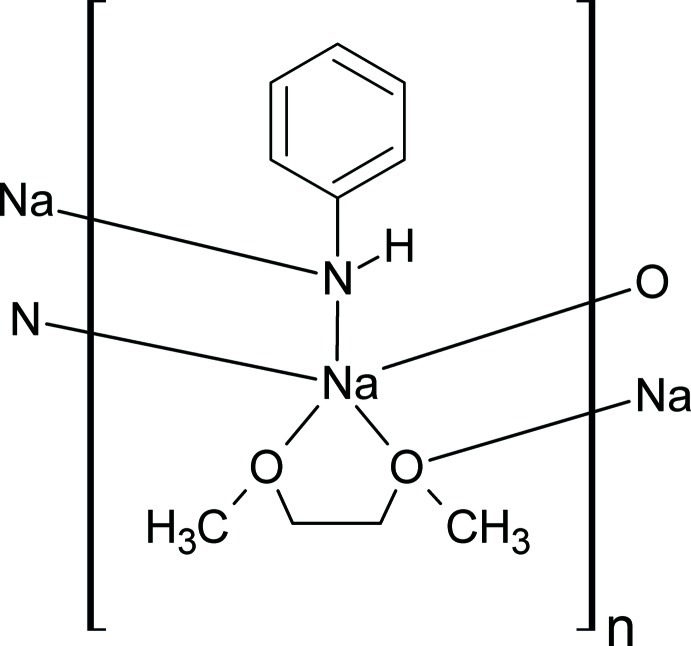


## Experimental

### 

#### Crystal data

[Na(C_6_H_6_N)(C_4_H_10_O_2_)]
*M* *_r_* = 205.23
Monoclinic, 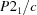

*a* = 7.0450 (7) Å
*b* = 13.1609 (14) Å
*c* = 12.5545 (12) Å
β = 101.153 (8)°
*V* = 1142.1 (2) Å^3^
*Z* = 4
Mo *K*α radiation
μ = 0.11 mm^−1^
*T* = 200 K
0.40 × 0.24 × 0.24 mm

#### Data collection

Stoe IPDS2T diffractometer
5398 measured reflections
2206 independent reflections
1450 reflections with *I* > 2σ(*I*)
*R*_int_ = 0.050

#### Refinement

*R*[*F* ^2^ > 2σ(*F* ^2^)] = 0.033
*wR*(*F* ^2^) = 0.078
*S* = 0.85
2206 reflections
131 parameters
H atoms treated by a mixture of independent and constrained refinement
Δρ_max_ = 0.14 e Å^−3^
Δρ_min_ = −0.18 e Å^−3^

### 

Data collection: *X-AREA* (Stoe & Cie, 2009 ▶); cell refinement: *X-AREA*; data reduction: *X-RED* (Stoe & Cie, 2009 ▶); program(s) used to solve structure: *SHELXS97* (Sheldrick, 2008 ▶); program(s) used to refine structure: *SHELXL97* (Sheldrick, 2008 ▶); molecular graphics: *DIAMOND* (Brandenburg, 2009 ▶); software used to prepare material for publication: *SHELXL97* and *PLATON* (Spek, 2009 ▶).

## Supplementary Material

Crystal structure: contains datablock(s) I. DOI: 10.1107/S1600536812040500/wm2683sup1.cif

Structure factors: contains datablock(s) I. DOI: 10.1107/S1600536812040500/wm2683Isup2.hkl

Additional supplementary materials:  crystallographic information; 3D view; checkCIF report

## Figures and Tables

**Fig. 1.**
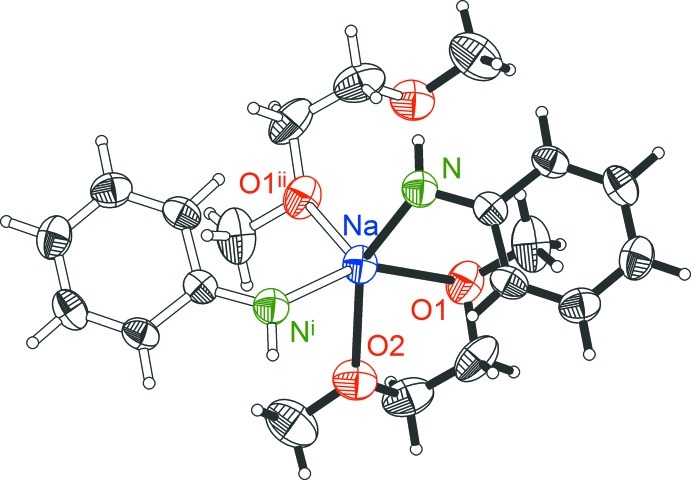
The coordination around the Na+ cation in the structure of the title compound. The asymmetric unit is marked by filled bonds. Anisotropic displacement parameters are drawn at the 50% probability level. [Symmetry codes: i: 1 - x, -y, 1 - z; ii: 2 - x, -y, 1 - z]

**Fig. 2.**
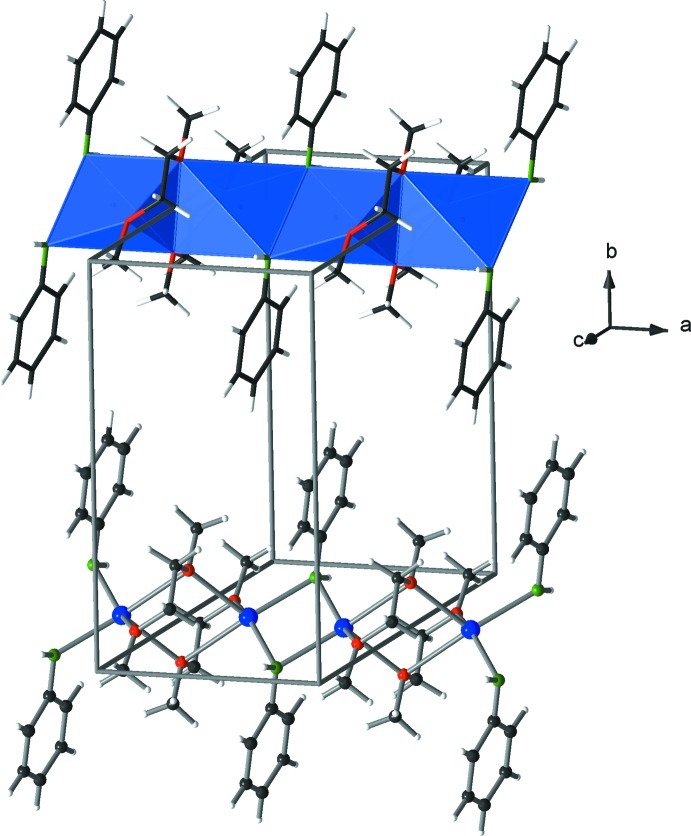
Part of the chain structure extending parallel to [100].

## supplementary crystallographic information

### Comment

In the title compound,[Na(C_6_H_5_NH)(C_4_H_10_O_2_)] or [Na(PhNH)(dme)], the Na^+^ cations are linked by bridging anilide (PhNH^-^) groups and µ_2_-κ^3^-*O,O*' coordinating 1,2-dimethoxyethane (dme) ligands to give a coordination polymer which extends parallel to [100]. The coordination around Na^+^ is distorted square pyramidal with two N atoms of the PhNH groups and two O atoms of the chelating dme ligands at the base of the square pyramid and a third O atom from an adjacing dme ligand at its apex (Fig.1). The Na—N distances of 2.4069 (14) and 2.4095 (11) Å are similar to the values that have been observed in the dimeric complex [{(pmdeta)Na(NHPh)}_2_] (2.390 (5) and 2.444 (5) Å, pmdeta = pentamethyldiethylenetriamine) (Barr *et al.*, 1995). Like in the case of the pmdeta derivative, the Na^+^ cations are linked by the PhNH groups to give planar Na_2_N_2_ rings with the phenyl groups in a *trans* arragement. The Na_2_N_2_ ring exhibits a rhombus shape with both the N—Na—N angle (93.22 (4)°) and the Na—N—Na angle (86.78 (4)°) close to 90°. A similar shaped Na_2_N_2_ ring has been observed in the pmdeta derivative (N—Na—N: 92.6°; Na—N—Na: 87.4°). The phenyl rings display a nearly perpedicular orientation with respect to the Na_2_N_2_ plane (82.16 (5)°). In the case of the pmdeta derivate a larger tilting (65°) due to the steric requirements of the pmdeta ligands is observed. In addition to the µ-bridging PhNH units, the Na^+^ cations are linked by µ_2_-κ^3^-*O,O*' coordinating dme ligands. The chelate ring formed by the atoms Na, O1, C8, C9 and O2 adopts an envelope conformation with the carbon atom C9 at the flap position. The Na—O distances within the chelate ring are 2.4103 (10) and 2.6800 (12) Å and the Na—O distance to the neighbouring Na^+^ cation amounts to 2.4849 (12) Å. Sodium complexes with similar µ-bridging dme ligands are known, *e.g.* with CCDC (Allen, 2002) reference code JAXQUU (Na—O: 2.348–2.461 Å; Bock *et al.*, 1999), WOTWAD (Na—O: 2.290–2.438 Å; Bock *et al.*, 2000), HUPZOH (Na—O: 2.371–2.668 Å; Tirla *et al.*, 2002) and QIBKIW (Na—O: 2.345–2.516 Å; Rothenberger *et al.*, 2007). Due to the µ-bridging mode of the dme molecules four membered Na_2_O_2_ rings possessing crystallographic 1 symmetry are formed. The Na_2_N_2_ and Na_2_O_2_ rings are linked by corner sharing to give an infinite chain parallel to [100]. The tilt angle between the Na_2_N_2_ and Na_2_O_2_ rings is 72.33 (4)°. Using a polyhedral model, the chain structure can be described by edge-sharing NaO_3_N_2_ square pyramids (Fig. 2). Interestingly, the H atom of the anilide NH group is not involved in hydrogen bonding.

### Experimental

0.7 ml (7.7 mmol) of aniline were added dropwise to a suspension of 0.29 g (7.5 mmol) of sodium amide in 10 ml of 1,2-dimethoxyethane. After one hour of stirring, the pale yellow reaction solution was reduced to 8 ml and afterwards layered with 20 ml of n-hexane. Colourless crystals of [Na(PhNH)(dme)] are formed at the phase boundary after one week. The product was filtered off and washed with n-pentane. Yield: 1.11 g (72%).

### Refinement

The H atom bonded to N was located from a difference Fourier map and was refined freely. Hydrogen atoms attached to the phenyl group and hydrogen atoms of the dme ligand were positioned geometrically and refined using a riding model with U(H) = 1.20 *U*_eq_(C).

### Crystal data

**Table:**

[Na(C_6_H_6_N)(C_4_H_10_O_2_)]	*F*(000) = 440
*M**_r_* = 205.23	*D*_x_ = 1.194 Mg m^−^^3^
Monoclinic, *P*2_1_/*c*	Mo *K*α radiation, λ = 0.71073 Å
Hall symbol: -P 2ybc	Cell parameters from 3804 reflections
*a* = 7.0450 (7) Å	θ = 4.0–29.2°
*b* = 13.1609 (14) Å	µ = 0.11 mm^−^^1^
*c* = 12.5545 (12) Å	*T* = 200 K
β = 101.153 (8)°	Prism, colourless
*V* = 1142.1 (2) Å^3^	0.40 × 0.24 × 0.24 mm
*Z* = 4	

### Data collection

**Table:**

Stoe IPDS2T diffractometer	1450 reflections with *I* > 2σ(*I*)
Radiation source: fine-focus sealed tube	*R*_int_ = 0.050
Graphite monochromator	θ_max_ = 26.0°, θ_min_ = 4.0°
Detector resolution: 6.67 pixels mm^-1^	*h* = −8→8
phi rotation scans	*k* = −14→16
5398 measured reflections	*l* = −15→15
2206 independent reflections	

### Refinement

**Table:**

Refinement on *F*^2^	Primary atom site location: structure-invariant direct methods
Least-squares matrix: full	Secondary atom site location: difference Fourier map
*R*[*F*^2^ > 2σ(*F*^2^)] = 0.033	Hydrogen site location: inferred from neighbouring sites
*wR*(*F*^2^) = 0.078	H atoms treated by a mixture of independent and constrained refinement
*S* = 0.85	*w* = 1/[σ^2^(*F*_o_^2^) + (0.0466*P*)^2^] where *P* = (*F*_o_^2^ + 2*F*_c_^2^)/3
2206 reflections	(Δ/σ)_max_ < 0.001
131 parameters	Δρ_max_ = 0.14 e Å^−^^3^
0 restraints	Δρ_min_ = −0.18 e Å^−^^3^

### Special details

**Table:**

Geometry. All e.s.d.'s (except the e.s.d. in the dihedral angle between two l.s. planes) are estimated using the full covariance matrix. The cell e.s.d.'s are taken into account individually in the estimation of e.s.d.'s in distances, angles and torsion angles; correlations between e.s.d.'s in cell parameters are only used when they are defined by crystal symmetry. An approximate (isotropic) treatment of cell e.s.d.'s is used for estimating e.s.d.'s involving l.s. planes.
Refinement. Refinement of *F*^2^ against ALL reflections. The weighted *R*-factor *wR* and goodness of fit *S* are based on *F*^2^, conventional *R*-factors *R* are based on *F*, with *F* set to zero for negative *F*^2^. The threshold expression of *F*^2^ > σ(*F*^2^) is used only for calculating *R*-factors(gt) *etc*. and is not relevant to the choice of reflections for refinement. *R*-factors based on *F*^2^ are statistically about twice as large as those based on *F*, and *R*- factors based on ALL data will be even larger.

### Fractional atomic coordinates and isotropic or equivalent isotropic displacement parameters (Å^2^)

**Table:**

	*x*	*y*	*z*	*U*_iso_*/*U*_eq_	
C1	0.53257 (15)	0.18227 (10)	0.39471 (10)	0.0352 (3)	
C2	0.53896 (17)	0.23456 (11)	0.49422 (10)	0.0419 (3)	
H2A	0.5200	0.1980	0.5547	0.050*	
C3	0.5719 (2)	0.33670 (12)	0.50437 (11)	0.0496 (4)	
H3A	0.5746	0.3676	0.5713	0.060*	
C4	0.6015 (2)	0.39515 (12)	0.41776 (13)	0.0543 (4)	
H4A	0.6253	0.4645	0.4255	0.065*	
C5	0.5944 (2)	0.34720 (12)	0.31930 (11)	0.0511 (4)	
H5A	0.6137	0.3851	0.2598	0.061*	
C6	0.55974 (18)	0.24503 (11)	0.30740 (10)	0.0420 (3)	
H6A	0.5538	0.2158	0.2394	0.050*	
C7	0.9515 (3)	−0.05477 (18)	0.79319 (15)	0.0823 (6)	
H7C	0.8362	−0.0954	0.7803	0.099*	
H7B	0.9620	−0.0198	0.8612	0.099*	
H7A	1.0623	−0.0978	0.7956	0.099*	
C8	1.1104 (2)	0.08004 (15)	0.72251 (13)	0.0651 (5)	
H8B	1.2216	0.0402	0.7127	0.078*	
H8A	1.1365	0.1079	0.7954	0.078*	
C9	1.0766 (2)	0.16376 (13)	0.64172 (14)	0.0637 (5)	
H9B	0.9640	0.2027	0.6510	0.076*	
H9A	1.1874	0.2090	0.6531	0.076*	
C10	1.0256 (2)	0.20221 (14)	0.45538 (16)	0.0686 (5)	
H10C	0.9439	0.2547	0.4747	0.082*	
H10B	0.9684	0.1749	0.3856	0.082*	
H10A	1.1504	0.2301	0.4523	0.082*	
N	0.50434 (16)	0.08063 (9)	0.38965 (10)	0.0446 (3)	
H1	0.505 (2)	0.0608 (12)	0.3207 (13)	0.046 (4)*	
O1	1.04685 (14)	0.12315 (8)	0.53495 (8)	0.0527 (3)	
O2	0.94293 (14)	0.01729 (9)	0.70809 (8)	0.0569 (3)	
Na	0.73912 (7)	0.00248 (4)	0.53096 (4)	0.04248 (17)	

### Atomic displacement parameters (Å^2^)

**Table:**

	*U*^11^	*U*^22^	*U*^33^	*U*^12^	*U*^13^	*U*^23^
C1	0.0227 (5)	0.0435 (8)	0.0375 (7)	0.0040 (5)	0.0011 (5)	0.0051 (6)
C2	0.0345 (6)	0.0553 (9)	0.0358 (7)	0.0019 (6)	0.0068 (5)	0.0052 (6)
C3	0.0462 (7)	0.0561 (10)	0.0462 (8)	0.0064 (7)	0.0082 (6)	−0.0075 (7)
C4	0.0522 (8)	0.0395 (9)	0.0696 (10)	0.0042 (6)	0.0075 (7)	0.0007 (7)
C5	0.0512 (8)	0.0512 (10)	0.0506 (8)	0.0071 (7)	0.0088 (6)	0.0175 (7)
C6	0.0411 (7)	0.0499 (9)	0.0338 (6)	0.0068 (6)	0.0039 (5)	0.0053 (6)
C7	0.0701 (11)	0.1031 (17)	0.0689 (11)	0.0194 (11)	0.0017 (9)	0.0246 (11)
C8	0.0443 (8)	0.0836 (13)	0.0606 (9)	0.0040 (8)	−0.0066 (7)	−0.0207 (9)
C9	0.0436 (8)	0.0558 (10)	0.0871 (12)	−0.0060 (7)	0.0009 (8)	−0.0263 (9)
C10	0.0407 (8)	0.0578 (10)	0.1068 (13)	0.0106 (7)	0.0134 (8)	0.0208 (10)
N	0.0407 (6)	0.0454 (7)	0.0454 (7)	0.0009 (5)	0.0028 (5)	0.0025 (6)
O1	0.0434 (5)	0.0450 (6)	0.0671 (6)	0.0064 (4)	0.0041 (4)	−0.0024 (5)
O2	0.0428 (5)	0.0686 (8)	0.0545 (6)	0.0079 (5)	−0.0025 (4)	0.0028 (5)
Na	0.0315 (2)	0.0423 (3)	0.0507 (3)	0.0027 (2)	0.0008 (2)	0.0036 (2)

### Geometric parameters (Å, º)

**Table:**

C1—N	1.3522 (18)	C8—C9	1.485 (3)
C1—C6	1.4147 (19)	C8—H8B	0.9700
C1—C2	1.4193 (19)	C8—H8A	0.9700
C1—Na	3.1112 (13)	C9—O1	1.4205 (19)
C2—C3	1.366 (2)	C9—H9B	0.9700
C2—H2A	0.9300	C9—H9A	0.9700
C3—C4	1.380 (2)	C10—O1	1.430 (2)
C3—H3A	0.9300	C10—H10C	0.9600
C4—C5	1.380 (2)	C10—H10B	0.9600
C4—H4A	0.9300	C10—H10A	0.9600
C5—C6	1.370 (2)	N—Na^i^	2.4069 (14)
C5—H5A	0.9300	N—Na	2.4095 (11)
C6—H6A	0.9300	N—H1	0.905 (16)
C7—O2	1.421 (2)	O1—Na^ii^	2.4849 (12)
C7—H7C	0.9600	O1—Na	2.6800 (12)
C7—H7B	0.9600	O2—Na	2.4103 (10)
C7—H7A	0.9600	Na—N^i^	2.4069 (14)
C8—O2	1.423 (2)	Na—O1^ii^	2.4849 (12)
			
N—C1—C6	125.59 (13)	H9B—C9—H9A	108.2
N—C1—C2	120.02 (13)	O1—C10—H10C	109.5
C6—C1—C2	114.39 (13)	O1—C10—H10B	109.5
N—C1—Na	47.33 (6)	H10C—C10—H10B	109.5
C6—C1—Na	138.68 (9)	O1—C10—H10A	109.5
C2—C1—Na	87.30 (7)	H10C—C10—H10A	109.5
C3—C2—C1	122.35 (13)	H10B—C10—H10A	109.5
C3—C2—H2A	118.8	C1—N—Na^i^	122.70 (10)
C1—C2—H2A	118.8	C1—N—Na	108.29 (8)
C2—C3—C4	121.59 (14)	Na^i^—N—Na	86.78 (4)
C2—C3—H3A	119.2	C1—N—H1	107.6 (10)
C4—C3—H3A	119.2	Na^i^—N—H1	113.5 (10)
C3—C4—C5	117.78 (15)	Na—N—H1	116.9 (9)
C3—C4—H4A	121.1	C9—O1—C10	111.21 (13)
C5—C4—H4A	121.1	C9—O1—Na^ii^	125.19 (8)
C6—C5—C4	121.41 (14)	C10—O1—Na^ii^	103.87 (10)
C6—C5—H5A	119.3	C9—O1—Na	102.22 (9)
C4—C5—H5A	119.3	C10—O1—Na	116.31 (8)
C5—C6—C1	122.46 (13)	Na^ii^—O1—Na	98.06 (4)
C5—C6—H6A	118.8	C7—O2—C8	112.21 (12)
C1—C6—H6A	118.8	C7—O2—Na	124.81 (11)
O2—C7—H7C	109.5	C8—O2—Na	119.72 (10)
O2—C7—H7B	109.5	N^i^—Na—N	93.22 (4)
H7C—C7—H7B	109.5	N^i^—Na—O2	89.99 (4)
O2—C7—H7A	109.5	N—Na—O2	147.82 (5)
H7C—C7—H7A	109.5	N^i^—Na—O1^ii^	111.25 (5)
H7B—C7—H7A	109.5	N—Na—O1^ii^	114.40 (4)
O2—C8—C9	108.92 (11)	O2—Na—O1^ii^	93.98 (4)
O2—C8—H8B	109.9	N^i^—Na—O1	154.19 (4)
C9—C8—H8B	109.9	N—Na—O1	101.45 (4)
O2—C8—H8A	109.9	O2—Na—O1	66.39 (4)
C9—C8—H8A	109.9	O1^ii^—Na—O1	81.94 (4)
H8B—C8—H8A	108.3	N^i^—Na—C1	106.16 (4)
O1—C9—C8	109.89 (13)	N—Na—C1	24.37 (4)
O1—C9—H9B	109.7	O2—Na—C1	125.28 (4)
C8—C9—H9B	109.7	O1^ii^—Na—C1	124.92 (4)
O1—C9—H9A	109.7	O1—Na—C1	81.39 (3)
C8—C9—H9A	109.7		
			
N—C1—C2—C3	178.31 (12)	C8—O2—Na—O1^ii^	80.20 (11)
C6—C1—C2—C3	−1.05 (17)	C7—O2—Na—O1	−157.16 (14)
Na—C1—C2—C3	142.48 (12)	C8—O2—Na—O1	0.80 (11)
C1—C2—C3—C4	−0.1 (2)	C7—O2—Na—C1	143.47 (13)
C2—C3—C4—C5	0.7 (2)	C8—O2—Na—C1	−58.57 (12)
C3—C4—C5—C6	−0.1 (2)	C9—O1—Na—N^i^	−5.56 (14)
C4—C5—C6—C1	−1.1 (2)	C10—O1—Na—N^i^	−126.90 (13)
N—C1—C6—C5	−177.64 (12)	Na^ii^—O1—Na—N^i^	123.21 (9)
C2—C1—C6—C5	1.67 (17)	C9—O1—Na—N	117.83 (9)
Na—C1—C6—C5	−114.27 (15)	C10—O1—Na—N	−3.51 (12)
O2—C8—C9—O1	−62.11 (17)	Na^ii^—O1—Na—N	−113.40 (5)
C6—C1—N—Na^i^	−135.23 (11)	C9—O1—Na—O2	−30.82 (8)
C2—C1—N—Na^i^	45.50 (13)	C10—O1—Na—O2	−152.15 (12)
Na—C1—N—Na^i^	98.16 (10)	Na^ii^—O1—Na—O2	97.96 (4)
C6—C1—N—Na	126.61 (11)	C9—O1—Na—O1^ii^	−128.78 (9)
C2—C1—N—Na	−52.66 (13)	C10—O1—Na—O1^ii^	109.89 (12)
C8—C9—O1—C10	−176.45 (12)	Na^ii^—O1—Na—O1^ii^	0.0
C8—C9—O1—Na^ii^	−50.41 (17)	C9—O1—Na—C1	103.91 (9)
C8—C9—O1—Na	58.76 (13)	C10—O1—Na—C1	−17.43 (11)
C9—C8—O2—C7	−170.51 (15)	Na^ii^—O1—Na—C1	−127.31 (4)
C9—C8—O2—Na	28.93 (17)	N—C1—Na—N^i^	−60.14 (12)
C1—N—Na—N^i^	123.46 (11)	C6—C1—Na—N^i^	−158.78 (13)
Na^i^—N—Na—N^i^	0.0	C2—C1—Na—N^i^	76.30 (8)
C1—N—Na—O2	28.35 (15)	C6—C1—Na—N	−98.64 (18)
Na^i^—N—Na—O2	−95.11 (9)	C2—C1—Na—N	136.44 (14)
C1—N—Na—O1^ii^	−121.44 (10)	N—C1—Na—O2	−161.95 (10)
Na^i^—N—Na—O1^ii^	115.10 (5)	C6—C1—Na—O2	99.41 (14)
C1—N—Na—O1	−35.19 (10)	C2—C1—Na—O2	−25.52 (9)
Na^i^—N—Na—O1	−158.65 (4)	N—C1—Na—O1^ii^	71.35 (11)
Na^i^—N—Na—C1	−123.46 (11)	C6—C1—Na—O1^ii^	−27.29 (15)
C7—O2—Na—N^i^	33.55 (14)	C2—C1—Na—O1^ii^	−152.21 (7)
C8—O2—Na—N^i^	−168.50 (11)	N—C1—Na—O1	145.16 (10)
C7—O2—Na—N	129.58 (14)	C6—C1—Na—O1	46.52 (14)
C8—O2—Na—N	−72.46 (14)	C2—C1—Na—O1	−78.40 (7)
C7—O2—Na—O1^ii^	−77.75 (13)		

Symmetry codes: (i) −*x*+1, −*y*, −*z*+1; (ii) −*x*+2, −*y*, −*z*+1.
